# Assessment of Tracheostomy Tube Placement and Late Change Practices in an Academic Tertiary Care Center

**DOI:** 10.1055/s-0043-1776723

**Published:** 2024-03-27

**Authors:** Christophe Abi Zeid Daou, Elsa Maria Chahine, Randa Barazi

**Affiliations:** 1 Department of Otolaryngology and Head and Neck Surgery, American University of Beirut Medical Center, Beirut, Lebanon; 2 American University of Beirut, Beirut, Lebanon

**Keywords:** tracheostomy, tube change, complications, pediatrics, prolonged intubation

## Abstract

**Introduction** The optimal time for tracheostomy changes is unknown. Most surgeons opt to wait until five to seven days postoperatively, while more recent studies suggest that changes occurring as early as two to three days postoperatively are also safe.

**Objective** To evaluate the safety of changing the tracheostomy tube later than 14 days postoperatively.

**Methods** The charts of patients who underwent tracheostomy placement and change at a tertiary care center from 2015 to 2019 were retrospectively reviewed, and the subjects were divided into 2 cohorts (late and very late), depending on the time of the first tracheostomy change.

**Results** The study included 198 patients, 53 of whom aged between 0 and 18 years, and 145, aged > 18 years. The time until the first tracheostomy change was on average of 131.1 days. The most common indication for tracheostomy tube placement was prolonged intubation. Adverse events were observed in 30.8% of the cases (the most common being the formation of granulation tissue), a rate that does not differ much from the incidence reported in the literature (of 34% to 77%) when tracheostomy tubes are changed as early as 3 to 7 days postoperatively. There was no significant difference in the incidence of complications between patients undergoing late and very late changes ( *p* = 0.688), or between pediatric and adult subjects ( *p* = 0.36). There were no significant correlations regarding the time of the first or second change and the incidence of complications (r = −0.014; *p* = 0.84 for the first change; and r = −0.57; *p* = 0.64 for the second change).

**Conclusion** The late first tracheostomy tube change was safe and could save resources and decrease the financial burden of frequent changes. It is always crucial to provide adequate information about home tracheostomy care for patients.

## Introduction

Tracheostomy is the surgical formation of an opening into the trachea. It is a procedure that has been performed for thousands of years – with the first reports of incisions into the “windpipe” dating from 1500 BC. The pioneers emphasized the crucial role of postoperative care, and the procedure has since gained popularity and standardization. The current indications for tracheostomy are numerous, and they mainly include the requirement of prolonged mechanical ventilation, upper airway obstruction, and the presence of copious secretions that are difficult to manage by the patients – putting them at risk of experiencing recurrent aspiration and developing pneumonia. 1 While the indications for tracheostomy are now standardized, guidelines regarding the long-term care of patients with tracheostomies are still lacking – with little to no evidence to guide the care and practice. 2

The insertion of a tracheostomy is associated with significant complications in the peri- and postoperative periods, especially in the pediatric population; 3 they include bleeding, accidental decannulation, cannula obstruction by mucous plugs or tracheostomy malfunction, the formation of granulation tissue and f fistulas, tracheomalacia, and wound breakdown. 4

Early changes of the tracheostomy tube are performed as soon as adequate maturation of the tracheostomy tract is accomplished and enables safe tube changes. 5 Late tracheostomy changes are usually avoided due to fear of increased infections, granulation, and complicated tube changes.

The time to change the tracheostomy tube and tracheostomy care practices are usually based on the preference of the surgeon and depend on institutional policies. 4

Tracheostomy tubes require change routinely for ongoing airway management and avoidance of long-term complications, such as infections and the formation of granulation tissue. 5 Clinically, the formation of granulation tissue can further complicate the care regarding a tracheostomy tube as it is prone to bleeding, and it can obstruct the tube and even delay decannulation attempts. 6 7 However, regular tube changes every two to three weeks have been shown to be effective in causing a dramatic decrease in the incidence of the formation of granulation tissue. Here again, the evidence is lacking as to the frequency of tracheostomy tube change – with different studies mentioning frequencies ranging from every 2 weeks to every 60 to 90 days. 5 6

The optimal time for tracheostomy tube changes is still unknown. Most surgeons opt to wait until five to seven days postoperatively, while more recent studies 8 studies suggest that changes occurring as early as two to three days postoperatively are also safe.

The American Academy of Otolaryngology-Head and Neck Surgery (AAO-HNS) published a consensus 9 that the first tracheostomy tube change should be performed after five to seven postoperative days to avoid procedure complications. However, in recent publications, 10 11 in the context of the coronavirus disease 2019 (COVID-19) pandemic, the AAO-HNS recommended delaying any tracheostomy changes until COVID-19 testing is negative to protect medical personnel. The statement was not supported by any formal studies.

Based on the financial burden of tracheostomy tube change and the fact that health insurers do not cover tracheostomy equipment and change, physicians were required to deviate from the published guidelines and perform the changes at later dates.

The purpose of the present study was to evaluate the safety of changing the tracheostomy tube later than 14 days postoperatively. The primary outcome is the development of any adverse event between the day of surgery and the first change.

## Methods

The present is a retrospective chart review study. It was approved by the Institutional Review Board (BIO-2019–0288) before any data from patients at the tertiary medical care center was accessed.

### Patient Selection

The study sample was composed of patients who underwent tracheostomy placement by an otolaryngologist at our tertiary care center from January 10, 2015, to July 30, 2019.

The inclusion criterion was patients who had undergone their first tracheostomy tube change at our institution. The exclusion criteria were patients with a history of airway surgery and those who had undergone their first tube change outside our institution. The patients were then divided into two cohorts according to the time of the first tube change: late change – patients who had undergone their first tracheostomy tube change before postoperative day 60; very late change – patients who had undergone their first tracheostomy tube change after postoperative day 60. A category for early change was not created because all changes had been performed at periods deemed at least “late”, and none had been performed according to the AAO-HNS consensus: after five to seven days postoperatively, .

### Surgical Technique

The surgical technique was the same for all pediatric patients, as well as for all adult patients in the present study. All insertions of tracheostomy tubes were performed surgically in the operating room (OR). No percutaneous tracheostomies were included.

Two residents perform the tracheostomy tube changes when they occur at the bedside or in the outpatient setting. The patient is put on supine position, with the neck extended. Scoping is performed through the tracheostomy tube to assess the airway. The tube is then removed and changed using the introducer at the bedside. The change is performed in the OR in case of suspicious findings on the scoping at the bedside (granulation tissue, tracheomalacia, abnormal anatomy), or if the patient cannot tolerate a bedside change (very young patient, those with multiple comorbidities). If the tube change is performed intraoperatively, then bronchoscopy is performed to rule out any airway/laryngeal pathology and then the tube is changed in the same manner as at the bedside.

### Data Recorded

Data included patient age at tracheostomy, the postoperative day when the tube was changed, postoperative complications, the location of the change, and whether the patients were decannulated during that change. The indications for tracheostomy were also noted for every patient. The sample was then divided into pediatric patients (aged between 0 and 18 years) and adults (aged >18 years) for a subanalysis.

### Statistical Analysis

A two-sample *t* -test was used to compare the differences between the late and very late changes regarding the frequency of complications, the location of the change, and decannulation. The Pearson correlation was used to determine the links between time of tube change and the prevalence of complications. The same was done when the sample was divided into pediatric and adult patients.

## Results

### Demographics

During the chart review, we identified 592 patients who underwent tracheostomy during the study, and 198 of them, 119 male and 79 female patients, were eligible for study inclusion ( Table 1 ). The average age at tracheostomy was of 45 **±** 29.6 years, with a minimum of 3 months and a maximum of 95 years. The time until the first tube change was on average of 131.1 ± 126.2 days, with a minimum of 14 days and a maximum of 540 days (∼ 1.5 years). The late change (14–60 days) group was composed of 92 patients (47%) and the very late change (after 60 days) group, of 106 patients (53%). During the first tube change, 20% (40) of the patients were decannulated.

**Table 1:** Demographics and characteristics of the overall sample

Category	Number of patients (% of total)	Mean(±standard deviation)
Gender		
Male	119 (60%)	
Female	79 (40%)	
Age at tracheostomy, in years		45.3(±29.6)
Time until tracheostomy tbe change (in days)		131.1(±126.2)
Time of tracheostomy tube change		
Late (14–60 days)	92 (47%)	
Very late (> 60 days)	106 (53%)	
Decannulation at first change	40 (20%)	
Late (14–60 days)	32 (80%)	
Very late (> 60 days)	8 (20%)	
Location of first change		
Operating Room	56 (28%)	
Intensive Care Unit	50 (26%)	
Emergency Room	24 (12%)	
Clinic	68 (34%)	
Patients who underwent second change	75 (38%)	
Time until second change (from first)		157.1(±179.4)
Decannulation at second change	20 (27%)	
Location of first change		
Operating Room	16 (21%)	
Intensive Care Unit	11 (15%)	
Emergency Room	22 (29%)	
Clinic	26 (35%)	

### Indications

**Fig. 1:**
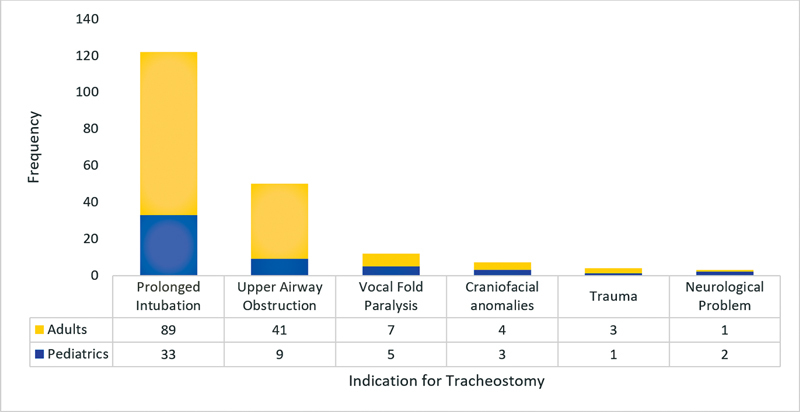
Indications for tracheostomy placement.

The most common indication for tracheostomy tube placement was prolonged intubation (62%), followed by upper airway obstruction (25%), vocal fold paralysis (6%), craniofacial anomalies (4%), trauma (2%), and neurological problems (1%) ( Fig. 1 ).

When dividing the sample between adult and pediatric patients, prolonged intubation was still the most common indication for tracheostomy. Upper airway obstruction was higher among adults (28%) than among children (17%), being the second most common indication for both groups of patients.

### First Tracheostomy Tube Change

In total, 53 patients were aged between 3 months and 18 years, with 11 (21%) undergoing decannulation at the first change (64% during a late change, 36% during a very late change; Table 2 ). The mean time until the change in the pediatric group was of 114.3 ± 111.2 days, with a minimum of 14 days and a maximum of 330 days. The changes were late in 57% of the pediatric group. The adult group was composed of 145 subjects, with 29 patients (20%) undergoing decannulation at the first change (86% during a late change and 14% during a very late change). The mean time until change in the adult group was of 137.2 ± 131.1 days, with a minimum of 14 days and a maximum of 540 days. The changes were late in 43% of the adult group. There were no significant differences between the two groups on terms of the time until the tube change ( *p* = 0.09).

**Table 2:** Demographics and characteristics of pediatric and adult patients

Category	Pediatric population	Adult population
Gender: n (%)		
Male	35 (66%)	84 (58%)
Female	18 (34%)	61 (42%)
Age at tracheostomy, in years: mean(±standard deviation)	4.35(±5.59)	60.34(±18.50)
Time until tracheostomy change, in days: mean(±standard deviation)	114.3(±111.2)	137.2(±131.1)
Time of tracheostomy change: n (%)		
Late (14–60 days)	30 (57%)	62 (43%)
Very late (> 60 days)	23 (43%)	83 (57%)
Decannulation at first change: n (%)	11 (21%)	29 (20%)
Late (14–60 days)	7 (64%)	25 (86%)
Very late (> 60 days)	4 (36%)	4 (14%)
Location of first change: n (%)		
Operating Room	25 (47%)	31 (21%)
Intensive Care Unit	14 (27%)	36 (25%)
Emergency Room	9 (17%)	15 (10%)
Clinic	5 (9%)	63 (44%)
Patients who underwent second change: n (%)	24 (45%)	51 (35%)
Time until second change (from first): n (%)	125.0 (±115.9)	173.8 (±204.5)
Decannulation at second change: n (%)	2 (8%)	11 (22%)
Location of first change: n (%)		
Operating Room	10 (42%)	6 (12%)
Intensive Care unit	6 (25%)	5 (10%)
Emergency Room	5 (21%)	17 (33%)
Clinic	3 (12%)	23(45%)

### Second Change

In total, 75 patients (38%) underwent second tube change, which occurred on average 157.1 ± 179.4 days after the first change, with a minimum of 2 days and maximum of 720 days; in 27% of these changes the patients were decannulated, and they mostly occurred in the clinic (35%).

On average, pediatric second changes were performed 125.0 ± 115.9 days after the first change, and adult second changes, 173.8 ± 204.5 days after the first change. Most of the pediatric changes were performed in the OR (42%), and adult changes, in the clinic (45%).

### Complications

**Fig. 2:**
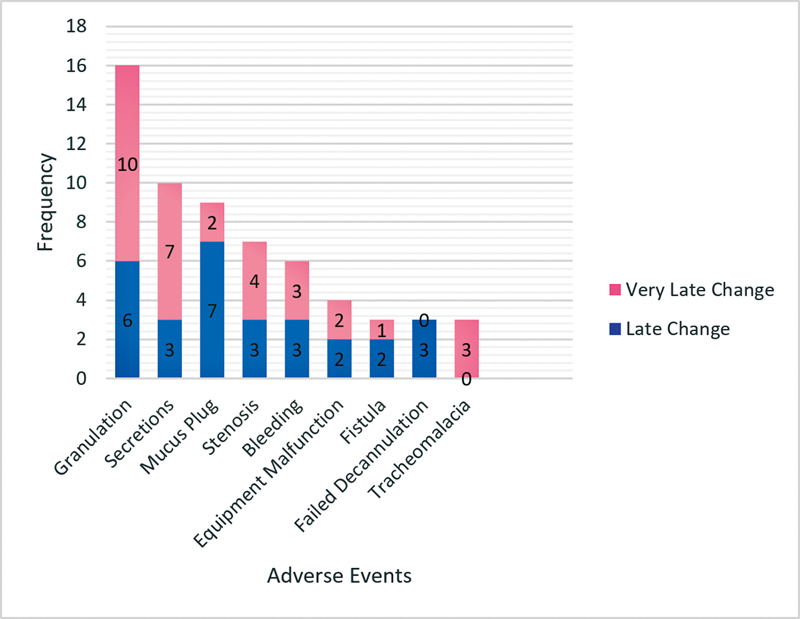
Frequency of adverse events at first tracheostomy tube change.

Of the 198 first tube changes performed, adverse events were observed in 30.8% (61) of the cases. In the pediatric population, the incidence was of 35.8%, and, in the adult population, of 28.9%. The most common adverse event was the formation of granulation tissue (8.1%), followed by heavy secretions (5.1%), cannula obstruction by mucus plug (4.5%), tracheal stenosis (3.5%), bleeding (3%), and equipment malfunction (2%). The least encountered complications were fistula formation, tracheomalacia and failed decannulation (1.5%) ( Fig. 2 ). There was no significant difference in the incidence of complications between the late and very late groups ( *p* = 0.688), nor between the pediatric and adult population ( *p* = 0.36).

There was no significant correlation between the time of the first tube change and the incidence of complications postoperatively (r = −0.014; *p* = 0.84), nor when comparing pediatric (r = −0.099; *p* = 0.48) and adult (r = 0.029; *p* = 0.29) first tube changes. Decannulation at the first change was significantly associated with fewer complications postoperatively (r = −0.227; *p* = 0.01).

During the second change, complications were observed in 40% of the cases (30/75). The most common adverse event was also granulation (5.1%), followed by heavy secretions (3.5%).

Neither were there significant differences in the incidence of complications between patients undergoing late and very late second changes ( *p* = 0.395), and no significant correlation was found between the time of the second change and the incidence of complications postoperatively (r = −0.57, *p* = 0.64).

Of the 75 patients undergoing second change, 40% experienced an associated complication, and 60% experienced none; 70% of the patients who experienced complications in the second change also experienced them in the first change, and 60% of the patients who did not experience complications in the second change did not experience them in the first change either.

The odds ratio of experiencing a certain adverse event during a second tube change when an adverse event was experienced during the first change was of 3.5 (95% confidence interval [95%CI] = 1.31–9.35)

### Location of Change

Most pediatric tube changes were performed in the OR (47%), while most changes in adults occurred at the clinic (44%). The location of the change was significantly different between pediatric and adult patients ( *p* < 0.001).

**Fig. 3:**
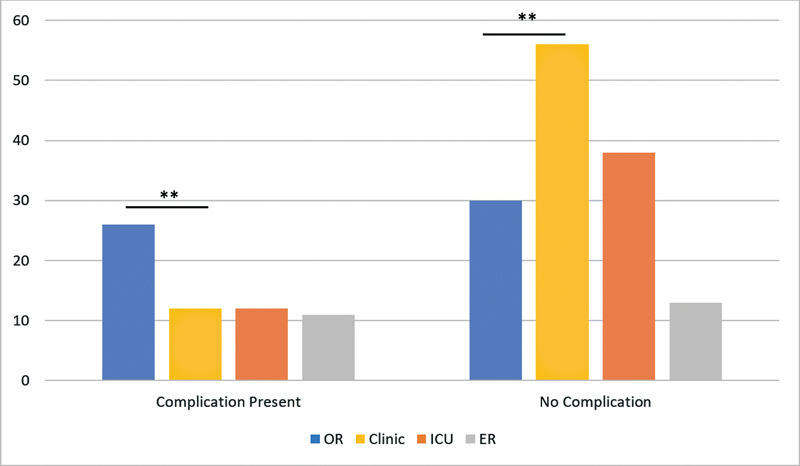
Prevalence of complications per location of tracheostomy tube change.

The prevalence of complications was the highest during changes in the OR (42.6%), followed by the Intensive Care Unit (ICU) and the clinic (19.7%), and the emergency room (18%). The prevalence of complications during a first change in the OR was significantly higher than that of the changes in the clinic ( *p* = 0.001), but not significant regarding any other setting ( Fig. 3 ). The incidence of complications during the second change was not correlated with the location of the change ( *p* = 0.204).

### Comparison with Pertinent Literature

The results of the present study were compared with those reported in the literature to assess the risks of late tube changes. The comparison is further detailed in the discussion section and in Table 3 .

**Table 3:** Comparison of our results with the pertinent results in the literature regarding complications and safety

Author	Date of publication	Pediatric (P) or adult (A) study population and sample size	Date of first tracheostomy change	Results	Results of the present study
Deutsch	1998	P, 21	Day 3–4	No major complications	No major complications; no deaths; AD: 0%; complication rate: 30.8%; GTF: 8.1%; Bleedong: 3%; Plug formation: 4.5%; WB: not available
Carron et al. 14	2000	P, 197	Day 5–7	Death rate: 3.6%; complication rate: 44%; GTF > 30%; TCF: 31%	
Carr et al. 15	2001	P, 142	Day 2–11	Complication rate: 77%; serious complications requiring surgery: 43%; GTF: 26%; bleeding: 2.5%; WB: 3.5%	
Van Burren et al. 12	2014	P, 151	Days 3–5	Death rate: 5.9%; AD: 2.5%; GTF: 1.5%; plug formation: 4.6%; WB: 4.5%	
Woods	2019	P, 83	Day 2–39	No deaths; TCF: 79%	
Chorney et al. 8	2021	P, 16	Day 4–7	AD: 0%; WB: 83.3%	
Zebda et al. 4	2021	P, 46	Day 3–7	AD: 0%; WB: 19.6%	
Fernandez-Bussy et al. 16	2015	A	Day 7	Complication rate: 65%; bleeding: 5.7%; plug formation: 0–3.5%	

Abbreviations: AD, accidental decannulation; GTF, granulation tissue formation; TCF, tracheocutaneous fistula formation; WB, wound breakdown.

## Discussion

The primary purpose of the present study was to evaluate the safety of performing the initial 2 tracheostomy tube changes later than the usual 14 days. Our review of 53 pediatric and 145 adult patients revealed no accidental decannulations and no deaths related to tracheostomy tube change. These results are similar to those reported by Zebda et al., 4 who observed no accidental decannulations in early changes at days 3 to 4 and 5 to 7 in neonates. Our results show a lower incidence of accidental decannulations and change-related deaths (related to airway loss) compared with the early changes in the case series by Van Buren et al. 12 Our results also show a lower incidence of tracheostomy-related deaths compared with the rate reported in the literature 13 (of 5.9% when changed at 7 days or before).

The incidence of complications after tracheostomy varies in the literature. In a review of 204 pediatric tracheostomies, Carron et al. 14 reported a complication rate of 44% and a rate of 3.6% of tracheotomy-related deaths. The tubes in their study 14 were changed 5 to 7 days postoperatively. The most common complication was granulation, followed by fistula, stenosis, and tube plugging. A review 13 of the literature published throughout the past three decades reported an overall complication rate ranging from 41.3% to 63% in children. In the present study, the rate of complications among pediatric patients was of 35.8%, with no tracheostomy-related deaths.

In the study by Carr et al., 15 the charts of 142 tracheostomies performed in children were reviewed, and the results show 11% of complications before the first tube change (at days 2 to 11), and 63% of complications after. Moreover, 15% of the decannulation attempts were unsuccessful, and 43% of the patients experienced serious complications, involving airway loss or requiring a separate surgical procedure. The complication rate found by Carr et al. 15 is higher than the one reported in the present study, in which there were no failed decannulations and none of the patients required a separate surgical procedure to remedy a certain complication.

In a review with adult subjects, Fernandez-Bussy et al. 16 reported an incidence of bleeding of 5.7%, an an incidence of tube obstruction ranging from 0% to 3.5% of the cases; the incidence of late complications after 1 week was reported to be of 65%.

In the present study, we did not observe an increased rate of complications overall (30.8%), in the pediatric population (35.8%), nor in the adult group (28.9%); neither were there severe adverse events involving total tube occlusion or requiring a separate operative procedure. Moreover, the results further support the fact that pediatric tracheostomies present a relatively higher rate of complications 3 4 ( Table 3 ).

In line with the literature, the main complication observed in the present study was also the formation of granulation tissue, followed by cannula obstruction. The incidence of bleeding in the present study was of 3%, and that of tube obstruction, of 4.5%. The incidence of adverse events at an even later second change was of 40%, which was not higher than the rate reported in the literature. The high incidence of mucus plugging can be explained by the fact that most patients are discharged home, and tracheostomy care at home is dependent on patient/family compliance.

In the present study, most complications were occurred in the OR, which can be explained by the fact that these patients were requiring bronchoscopy due to complicated cases or very young age. Moreover, some patients were taken to the OR due to the complications, to undergo therapeutic intervention for a given adverse event, that is, laser ablation for granulation tissue or control of bleeding.

It is important to note that some studies 8 12 advocate early changes as they have been shown to decrease the length of ICU and hospital stays. However, it is not within the practices of our institution to wait for the first change for patients to be transferred or discharged. Tracheostomy care is only delayed for 24 hours postoperatively, after which the patient can be transferred or discharged, and sedation can be weaned off if medically allowed. Indeed, most of our patients were discharged home prior to their first tracheostomy change. Their change was performed in the outpatient setting (clinic or emergency room) or in subsequent hospitalizations.

Decannulating the patient at the first change also decreases morbidity. However, in patients not being decannulated, it is important to note incidence of adverse events on a given change, because the same patient is likely to have complications at subsequent changes too. This could help physicians better plan future changes, and maybe schedule them in the OR or in a controlled setting to further decrease morbidity and mortality.

The cost of a tracheostomy tube can range from US$50 to US$243. 17 The average cost of an adult tracheostomy tube is of US$50.11 (most commonly, a fenestrated cuffed tube is used). The average cost of a pediatric tube is of US$97.08. Changing a tracheostomy tube every 2 to 3 weeks, every 29 days on average, is recommeded. 5 6 This means that, within a year (365 days), the adult patient will pay US$601.32, and the pediatric patient, US$1,164.96 for 12 changes on average. The mean time until the change in the present study was of 131.1 days. Within 1 year, the adult patient in our institution will pay US$150.33, and the pediatric patient, US$291.24 for 3 changes on average. It is important to take into consideration that tracheostomy tubes are not covered by health insurances in certain countries, just as in ours, and less frequent changes relieve patients of the economic burden (costing four times less) without any added risk.

The limitations of the present study are related to the retrospective nature of the data collection. The recorded data are limited and might be incomplete. In that aspect, the rate of wound breakdown was not assessed due to limited data in the charts; however, even when present, it did not cause significant morbidity. There was no control group, as these patients were not randomized and no changes were performed earlier than 14 days to compare the results; therefore, we could only compare our data with that available in the literature. Future prospective studies with appropriate controls may provide further evidence to help guide physicians in the development of management guidelines.

## Conclusion

Late tracheostomy tube change, at a mean of 114.3 days in children and 137.2 days in adults, was safely performed in 198 patients. Routine late tube change in many patients could save resources and decrease the financial burden of frequent changes. Moreover, this late change was not associated with increased complications during subsequent changes.

It is crucial to provide adequate information about home tracheostomy care for patients and families to avoid postoperative adverse events. However, it is still important to tailor the management to the patient and time tube changes on a case basis while carefully choosing the setting of change in case bronchoscopy is needed. Based on our results, physicians can wait to perform tube changes, discharge, or transfer their patients, without the risk of increased complications or morbidity. However, when a complication is observed, it is important to keep it in mind when planning future changes for a given patient. More studies are needed to confirm our findings.
